# Acute Cholangitis Masquerading Biliary Neuroendocrine Carcinoma: A Rare Twist to a Typical Presentation

**DOI:** 10.14309/crj.0000000000001228

**Published:** 2023-12-16

**Authors:** Talwinder Nagi, Zoilo K. Suarez, Muhammad Adnan Haider, Charles Vallejo, Oscar Hernandez, Theodore Doukides

**Affiliations:** 1 Department of Internal Medicine, Florida Atlantic University, Schmidt College of Medicine, Boca Raton, FL; 2 Department of Gastroenterology, Florida Atlantic University, Schmidt College of Medicine, Boca Raton, FL

**Keywords:** biliary carcinoma, neuroendocrine carcinoma, cholangitis, CBD stenosis, ERCP

## Abstract

Biliary neuroendocrine carcinoma (BNEC) develops in the biliary tract and is characterized by the presence of neuroendocrine cells and account for less than 1% of all malignancies. We present a patient with no significant risk factors of BNEC who presented with abdominal pain and jaundice. An endoscopic ultrasound with endoscopic retrograde cholangiopancreatography was concerning for neoplasm and less likely Mirizzi syndrome. Pathology revealed well-differentiated grade 3 large-cell neuroendocrine carcinoma of the common bile duct. BNEC has a poor prognosis with a reported 5-year survival rate of less than 20%, which is worse than other biliary tract malignancies such as cholangiocarcinoma. Additional cases and further studies of multimodal treatment are required in the future to improve prognosis. Providers should be aware of the risk factors of BNEC and consider the diagnosis when evaluating patients with the symptoms above.

## INTRODUCTION

Biliary neuroendocrine carcinoma (BNEC) is a rare type of cancer that develops in the biliary tract and is characterized by the presence of neuroendocrine cells. Biliary tract cancers, in general, are uncommon, accounting for less than 1% of all malignancies.^1^ Among biliary tract cancers, BNEC is one of the rarest subtypes, with an incidence of less than 1 case per million people per year. Extrahepatic BNEC are rare and reportedly only account for 0.32% of primary neuroendocrine tumor sites.^2^ The most frequent sites of extrahepatic BNEC are the common hepatic duct and the distal common bile duct (19.2%), followed by the middle of the common bile duct (17.9%), the cystic duct (16.7%), and the proximal common bile duct (11.5%).^2^

The exact cause of BNEC is unknown. However, like most cancers, it is believed to result from a combination of genetic and environmental factors. Some risk factors associated with the development of BNEC include chronic inflammation of the biliary tract, liver cirrhosis, and infection with hepatitis B or C virus.

We present an 80-year-old man with no significant risk factors of BNEC, as described above, who presented with abdominal pain and jaundice.

## CASE REPORT

An 80-year-old man with a medical history of hypertension, hyperlipidemia, benign prostatic hyperplasia, and gastroesophageal reflux disease presented to the emergency department with a complaint of fever starting 8 hours ago. He endorsed poor oral intake and abdominal pain in the right upper quadrant that started 2 weeks ago associated with postprandial pain. He noticed yellowing of his skin and eyes, dark-colored urine 5 days earlier, and a 10-pound weight loss over the past few weeks. The patient denied any sick contacts, travel history, or previous gastrointestinal issues. He had mild nausea and vomiting, denied smoking, alcohol, or drug use.

**Figure 1.**
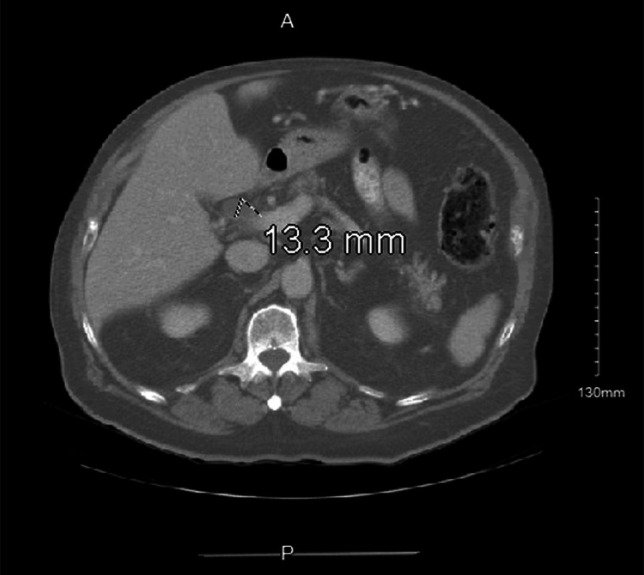
CT of abdomen and pelvis with IV and oral contrast depicting dilated CBD to 1.3 cm with an abrupt caliber change and concern for wall thickening. CBD, common bile duct; CT, computed tomography; IV, intravenous.

**Figure 2.**
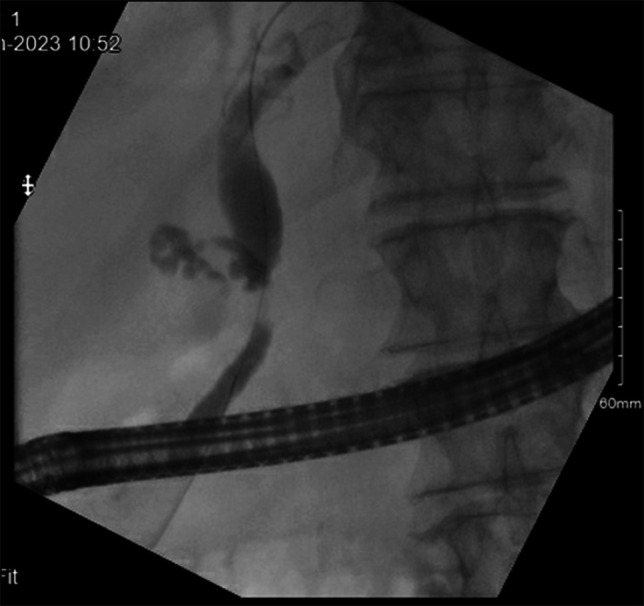
ERCP image demonstrating moderate-to-severe stenosis/stricture extending approximately 1.5 cm in the mid CBD located in the proximity of the cystic duct takeoff. CBD, common bile duct; ERCP, endoscopic retrograde cholangiopancreatography.

On evaluation in the emergency department, he was hemodynamically stable and afebrile. The patient was in no visible distress, overweight habitus, and appropriately alert and oriented. There was apparent bilateral scleral icterus with jaundiced and dry skin. The abdomen was nondistended with normal bowel sounds, and tenderness to palpation in the epigastric region was appreciated with no guarding or rigidity. The laboratory results were significant for elevated white blood cell count of 26.8 K/mcL, alkaline phosphatase of 494 IU/L, aspartate transaminase of 134 IU/L, alanine transaminase of 198 IU/L, and an elevated total bilirubin of 4.6 mg/dL with an elevated direct bilirubin of 2.4 mg/dL. Blood cultures were drawn, and he was started on intravenous piperacillin-tazobactam. A computed tomography (CT) of the abdomen and pelvis with intravenous and oral contrast showed no hepatic lesions, no intrahepatic biliary dilatation, a dilated common bile duct (CBD) to 1.3 cm with an abrupt caliber change and concern for wall thickening, and normal-appearing pancreas with no pancreatic ductal dilatation (Figure 1). Owing to the leukocytosis, subjective fevers, jaundice, and bile duct thickening with contrast enhancement, there was concern for acute cholangitis. An endoscopic ultrasound was completed which found sludge in the gallbladder but no gallbladder mass, and extrahepatic bile duct dilatation at the level of the confluence and common hepatic duct measuring up to 12 mm with abrupt caliber change in the mid bile duct at the level of the cystic duct takeoff. Bile duct wall thickening was noted. There was no regional lymphadenopathy, and the pancreatic duct and left lobe of the liver were unremarkable. A subsequent endoscopic retrograde cholangiopancreatography (ERCP) showed moderate-to-severe stenosis extending approximately 1.5 cm in the mid CBD (Figure 2). This was located in the proximity of the cystic duct takeoff. A 10-mm wire-guided biliary sphincterotomy was performed. Wire-guided SpyDS cholangioscopy was performed and demonstrated mucosal nodularity and neovascularization at the level of the stricture. No stones were visualized. Under direct visualization, SpyMax forceps biopsies were obtained of the stricture site and wire-guided brushings of the stricture for cytology. A 10 × 80 mm fully covered metal biliary stent was deployed with prompt drainage of contrast and purulent bilious fluid. Morphology by ERCP and cholangioscopy was concerning for neoplasm and less likely for Mirizzi syndrome. Given the findings on imaging and endoscopic intervention, the stricture was extrahepatic and extrapancreatic in nature. Subsequent laboratory results showed an elevated CA 19-9 of 55 units/mL. Immunohistochemistry completed for pathology on ERCP sampling of the CBD was used to determine that this is a neuroendocrine carcinoma. The atypical cells coexpressed cytokeratin cocktail AE1/AE3, synaptophysin, chromogranin, and CD56, which are specific for neuroendocrine tumors. Ki-67 proliferation rate was > 20% (range 20%–40%), which is suggestive of grade 3 of 3 tumor. He was taken to the operating room for resection of the common hepatic duct, CBD, cystic duct, hepatic hilar lymph node dissection, cholecystectomy, pancreaticoduodenectomy, and hepaticojejunostomy. The patient tolerated the procedure well and was managed in the surgical intensive care unit postoperatively. Final pathology from the operation revealed large-cell neuroendocrine carcinoma of the CBD invading into the periductal soft tissue to a depth of 4 mm with 18 negative lymph nodes. Two months after surgical resection, the patient completed a gallium 68 DOTA-TATE positron emission tomography-CT scan that showed no abnormal radiotracer accumulation. He had no evidence of metastatic disease. At a 6-month follow-up with hematology oncology, he was feeling well despite mild increase in frequency of bowel movements. Repeat CT imaging at 6 months showed no abnormal findings suspicious for recurrence.

## DISCUSSION

Owing to its rarity and diagnostic challenges, BNEC is often misdiagnosed or diagnosed at an advanced stage, leading to poor prognosis and limited treatment options. Neuroendocrine carcinomas, especially biliary, are slow growing malignant tumors without specific clinical manifestations, thus leading to risk of late diagnosis.^2^ As mentioned, BNEC usually presents with nonspecific symptoms such as abdominal pain, jaundice, nausea and vomiting, weight loss, fever, and fatigue. These symptoms can be attributed to other biliary tract tumors or other conditions such as the Mirizzi syndrome or acute cholangitis, leading to misdiagnosis and delayed treatment. BNEC can present asymptomatically and be detected incidentally during imaging studies. Only a few cases of BNEC have been reviewed in the literature with most patients presenting with complaints of jaundice and right upper quadrant pain.^2–5^ Similar to our patient, an 80-year-old man presented with jaundice, and the workup with ERCP showed complete obstruction of the CBD by a 1.5 cm mass. Interestingly, there were no malignant cells in the biliary brush cytology; however, the patient underwent extrahepatic bile duct resection because of suspicions of cholangiocarcinoma.^5^ Final histology and pathology confirmed large-cell neuroendocrine carcinoma; however, because of delayed detection, the patient died after 3 months from metastatic disease. One other case presented a 59-year-old man with a complaint of jaundice, and CT imaging showed a 4.5 cm mass involving the CBD.^4^ He underwent radical CBD and Roux-en-Y hepaticojejunostomy, and histology confirmed large-cell neuroendocrine carcinoma. This patient also received adjuvant chemotherapy and radiotherapy because of tumor invasion into proximal resection margins. No recurrence was detected on CT imaging at a 10-month follow-up.^4^ Surgical resection is the mainstay of treatment for BNEC, but the extent of surgery depends on the location and size of the tumor. For more advanced tumors, adjuvant chemotherapy and radiation may be necessary to improve chances of long-term survival. The choice of chemotherapy for BNEC is not well established, but some studies suggest that platinum-based chemotherapy may be effective. BNEC has a poor prognosis with a reported 5-year survival rate of less than 20%. This prognosis is worse than other biliary tract malignancies such as cholangiocarcinoma. An accumulation of additional cases and further studies of multimodal treatment are required in the future to improve prognosis. Patients who experience symptoms such as abdominal pain, jaundice, or weight loss should seek medical care because these symptoms may indicate the presence of BNEC. Healthcare providers should be aware of the risk factors and symptoms of BNEC and consider the diagnosis when evaluating patients with the symptoms above.

## DISCLOSURES

Author contributions: T. Nagi wrote the original manuscript and is the article guarantor. All authors reviewed and provided revisions to the manuscript.

Financial disclosure: None to report.

Informed consent was obtained for this case report.
